# GPR173 agonist phoenixin 20 promotes osteoblastic differentiation of MC3T3-E1 cells

**DOI:** 10.18632/aging.103717

**Published:** 2020-11-10

**Authors:** Zhengtao Gu, Denghui Xie, Rui Ding, Caiqiang Huang, Yiyan Qiu

**Affiliations:** 1 Department of Treatment Center for Traumatic Injuries, Guangdong Provincial Key Laboratory of Bone and Joint Degeneration Diseases, Academy of Orthopedics of Guangdong Province, The Third Affiliated Hospital of Southern Medical University, Guangzhou, Guangdong Province, China; 2 Division of Joint Surgery, Department of Orthopedics, Guangdong Provincial Key Laboratory of Bone and Joint Degeneration Diseases, Academy of Orthopedics of Guangdong Province, The Third Affiliated Hospital of Southern Medical University, Guangzhou, Guangdong Province, China; 3 Division of Spine Surgery, Section II, Department of Orthopedics, Academy of Orthopedics of Guangdong Province, The Third Affiliated Hospital of Southern Medical University, Guangzhou, Guangdong Province, China

**Keywords:** osteogenic differentiation, MC3T3-E1, GPR173, phoenixin 20

## Abstract

Osteogenic differentiation is critical to bone homeostasis, and its imbalance plays a key role in the progression of osteoporosis. Osteoblast cells are responsible for synthesizing new bone tissue, and understanding how to control osteoblastic differentiation is vital to the treatment of osteoporosis. Herein, we show that GPR173 signaling is involved in the regulation of osteoblastic differentiation in MC3T3-E1 cells. Our data reveals that GPR173 is abundantly expressed in MC3T3-E1 cells, and its expression is inducible upon the introduction of osteogenic media. The activation of GPR173 by its selective agonist phoenixin 20 induces the expression of several osteoblast signature genes including collagen type 1 alpha 1 (Col-I), osteocalcin (OCN), alkaline phosphatase (ALP) as well as increased matrix mineralization and ALP activity, suggesting that the activation of GPR173 promotes osteoblastic differentiation. Moreover, we show that the effect of phoenixin 20 is mediated by its induction on the key regulator runt-Related Transcription Factor 2 (Runx2). Mechanistically, we display that the action of phoenixin 20 requires the activation of MAPK kinase p38, and deactivation of p38 by its inhibitor SB203580 weakens the phoenixin 20-mediated induction of RUNX-2, ALP, and matrix mineralization. Silencing of GPR173 attenuates phoenixin 20-mediated osteoblastic differentiation, indicating its dependence on the receptor. Collectively, our study reveals a new role of GPR173 and its agonist phoenixin 20 in osteoblastic differentiation.

## INTRODUCTION

Osteoporosis is a condition in which bone becomes weak which is characterized as low bone mass and structural deterioration. As a result, bone tissue becomes fragile and shows increased vulnerability to fracture. World wide, osteoporosis affects about 200 million people and is often unrecognized until one encounters a fracture due to the silent nature of the disease [1]. Under healthy conditions, bone is maintained by the constant process of bone remodeling. Normal bone remodeling maintains a balance between bone resorption and formation to maintain bone density. Osteoblasts are generated from the osteogenic differentiation of mesenchymal stem cells (MSCs). MSCs possess the capacity to self-renew and to differentiate into multiple cell types. It has been known that MSCs are common precursors for osteoblasts [2]. The direction of MSC differentiation depends on specific regulatory factors. RUNX-2 acts as a key transcriptional modulator that mediates the conversion of MSCs to osteoblasts [3]. Runx2 is a bone-related transcription factor homologous to the Drosophila protein, Runt [4]. Runx2 is post-translationally modified (PTM) downstream of a diverse set of signaling pathways whose coordinated action controls osteoblast differentiation and bone development [5]. Genes associated with matric formation, remodeling, and mineralization have been reported to be regulated by RUNX2 [6]. Runx2 is regulated by the p38 MAPK pathway, which plays an important role in osteoblast differentiation and bone formation [7]. As osteoblasts mature, they begin producing the bone extracellular matrix by secreting bone matrix proteins, including collagen type 1 alpha 1 (Col-I), osteocalcin (OCN), and alkaline phosphatase (ALP). Type I collagen is the main constituent of bone, comprising the non-mineralized bone matrix. The accumulation of calcium phosphate in the bone matrix by ALP leads to the mineralization of bone. Mature osteoblasts express ALP, Col-I, osteocalcin, and osterix [8]. It has been recognized that the normal differentiation capacity of MSCs is altered in osteoporosis, thereby hindering osteoblast formation [9].

GPR173 belongs to a subfamily of G protein-coupled receptors and is also referred to as super conserved receptor expressed in brain (SREB). These receptors are highly conserved and uniquely expressed in central neural systems. GPR173, originally termed SREB3, is the most conserved receptor in this family as human GPR173 and rodent GPR173 share 99% of the same amino acids [10]. GPR173 has been found to be present in various tissues and organs and possesses a diverse range of biological functions. Now, several studies have linked GPR173 as the receptor of phoenixin, a newly identified hormone. Phoenixin has been reported to be involved in the modulation of memory formation, depression, reproduction, and food intake [11, 12]. Two members of the phoenixin family, phoenixin-14 amide and phoenixin-20 amide, which are comprised of 14 or 20 amino acid chains, respectively, have been isolated and identified. It has been shown that the hypothalamus is the tissue with the greatest expression of phoenixin, and phoenixin 20 is the predominant isoform of phoenixin [11]. Previous studies have reported pleiotropic roles of phoenixin in regulating sensory perception, body weight, reproduction, and the cardiovascular system [13]. For example, phoenixin plays an important role in the release of luteinizing hormone (LH) by augmenting the release of gonadotropin-releasing hormone (GnRH), which is mediated by GPR173 [14]. Although this peptide has been linked with the development of obesity, diabetes, cardiovascular diseases, anxiety, and depression, further investigations are helpful in specifying the physiological functions of phoenixin, and especially peripheral phoenixin. In this study, we aimed to investigate the biological function of GPR173 and its agonist phoenixin 20 in osteogenic differentiation. Our results indicate that the GPR173 agonist phoenixin 20 promotes osteoblastic differentiation mediated by the p38/RUNX-2 pathway.

## RESULTS

### GPR173 is expressed in osteoblast cells

**Figure 1:**
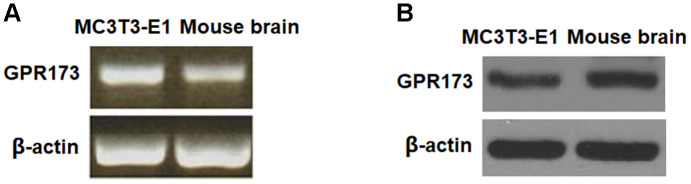
**GPR173 is expressed in MC3T3-E1 cells.** The expression of GPR173 was measured using mouse brain as a reference. (**A**) RT-PCR of GPR173; (**B**) Western blot of GPR173. Experiments were repeated for 3 times.

To clarify the function of GPR173 in osteogenic differentiation, we first assessed its expression profile in osteoblastic precursor cells MC3T3-E1. MC3T3-E1 cells are a pre-osteoblast cell line and possess the ability to differentiate into osteoblasts, which have been widely used for an ideal model for the differentiation, matrix deposition, and mineralization of osteoblastic cells [15]. We used mouse brain lysate as the reference, which is known to express GPR173. By RT-PCR, we revealed that the relative mRNA expression of GPR173 in MC3T3-E1 is comparable to its expression in brain tissue (Figure 1A). By western blot, we further confirmed the abundant expression of GPR173 as compared to its level in mouse brain (Figure 1B).

### GPR173 is induced for osteoblast differentiation

**Figure 2:**
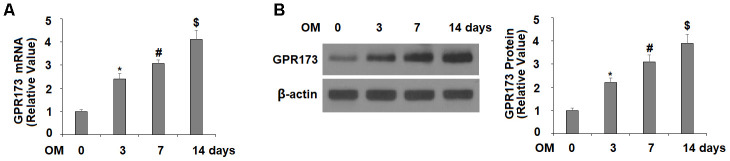
**Expression of GPR173 was elevated during osteoblast differentiation.** Cells were cultured with osteogenic medium for various times. (**A**) mRNA levels of GPR173; (**B**) Protein levels of GPR173 (*, #, $, P<0.01, n=4-5).

Next, we monitored the expression profile of GPR173 during osteoblastic differentiation of MC3T3-E1 cells. In our experiment, the cells were fed with osteogenic medium (OM) to induce osteoblastic differentiation. Our time-course experiment showed that the mRNA level of GPR173 was gradually induced up to about 4-fold after 3, 7, and 14 days of induction (Figure 2A). Meanwhile, the western blot experiment confirmed a similar trend of time-dependent induction of GPR173 over the same periods of time (Figure 2B).

### GPR173 agonist phoenixin 20 promotes osteoblastic differentiation

**Figure 3:**
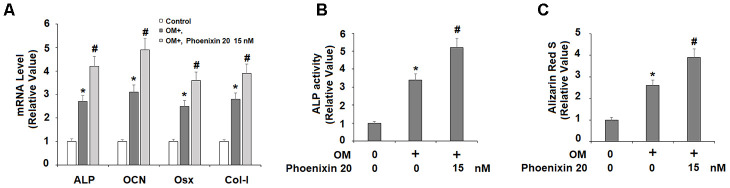
**Agonism of GPR173 with phoenixin 20 promoted osteoblast differentiation.** Cells were cultured with osteogenic medium (OM) with or without phoenixin 20 (15 nM) for 14 days. (**A**) Gene levels of ALP, OCN, Osx, and Col-I; (**B**) ALP activity; (**C**). Alizarin Red S staining (*, #, P<0.01, n=5).

The responsive induction of GPR173 suggests its potential role in osteoblastic differentiation. We then treated MC3T3-E1 cells with GPR173 selective agonist phoenixin 20 while they were fed with the osteogenic medium. We assessed the expression of four osteoblastic signature genes, including ALP, Col-I, Osx, and OCN. Compared to the non-treatment control group at day 14, the mRNA expression of ALP, OCN, Osx, and Col-I in the cells growing in osteogenic media plus phoenixin 20 were all several-fold higher than the cells maintained in osteogenic media alone (Figure 3A). We stained cells with Alizarin Red S to evaluate matrix mineralization in the differentiated MC3T3-E1 cells. Compared to the control group, the cells with phoenixin 20-supplemented osteogenic media at day 14 had a significant increase in matrix mineralization as compared to the cells without phoenixin 20 (Figure 3B). These data suggested that phoenixin accelerated the osteoblastic differentiation of MC3T3-E1 cells.

### Phoenixin 20 increases RUNX-2 expression for osteoblastic differentiation

**Figure 4:**
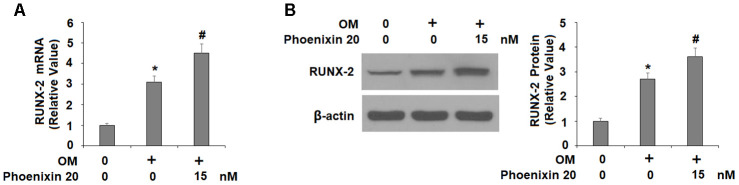
**Agonism of GPR173 with phoenixin 20 increased RUNX-2.** Cells were stimulated osteogenic medium with or without phoenixin 20 (15 nM) for 14 days. (**A**) mRNA of RUNX-2; (**B**) Protein of RUNX-2 (*, #, P<0.01, n=4).

To explore the molecular mechanism of GPR173-mediated osteoblastic differentiation, we examined several key regulators involved in this process. RUNX-2 is a critical transcription factor for modulating osteoblast differentiation, and thus, we assessed the expression of RUNX-2 during osteoblastic differentiation. In the same treatment experiment and compared to non-treated cells, 14 days of osteogenic medium induced about 3-fold RUNX-2 mRNA, but the presence of phoenixin 20 in the osteogenic medium increased RUNX-2 mRNA about 4.5-fold (Figure 4A). At the protein level, osteogenic medium induced about 2.7-fold RUNX-2 expression, but the presence of phoenixin 20 resulted in about 3.6-fold RUNX-2 (Figure 4B).

### Phoenixin 20 activates p38 for osteoblastic differentiation

**Figure 5:**
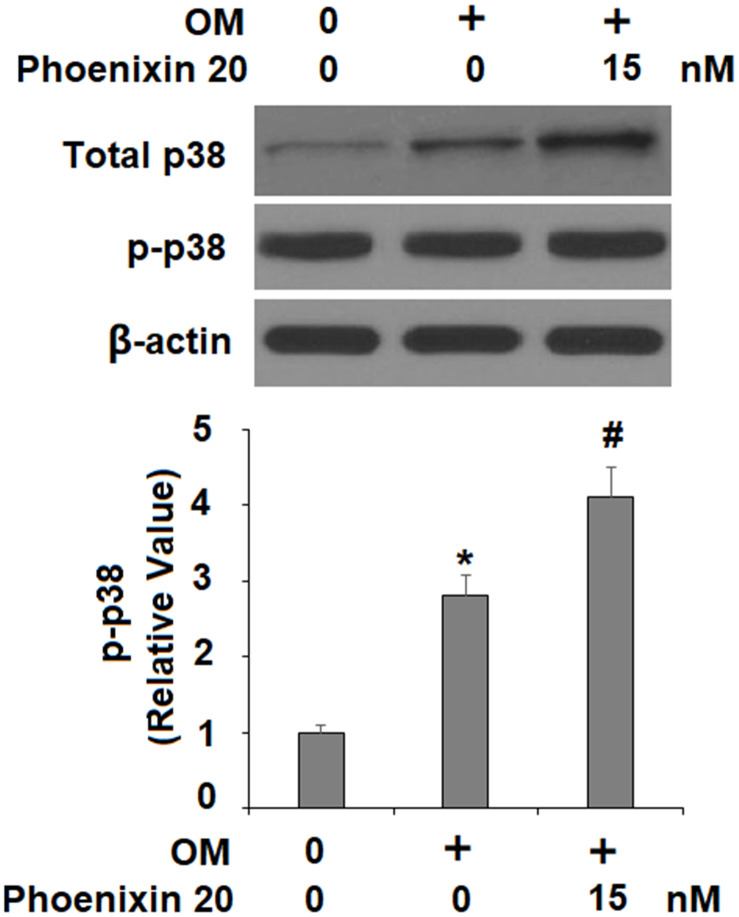
**Phoenixin 20 activates p38.** Cells were stimulated with osteogenic medium with or without phoenixin 20 (15 nM) for 2 h. Phosphorylated and total p38 were detected (*, #, P<0.01, n=4).

**Figure 6:**
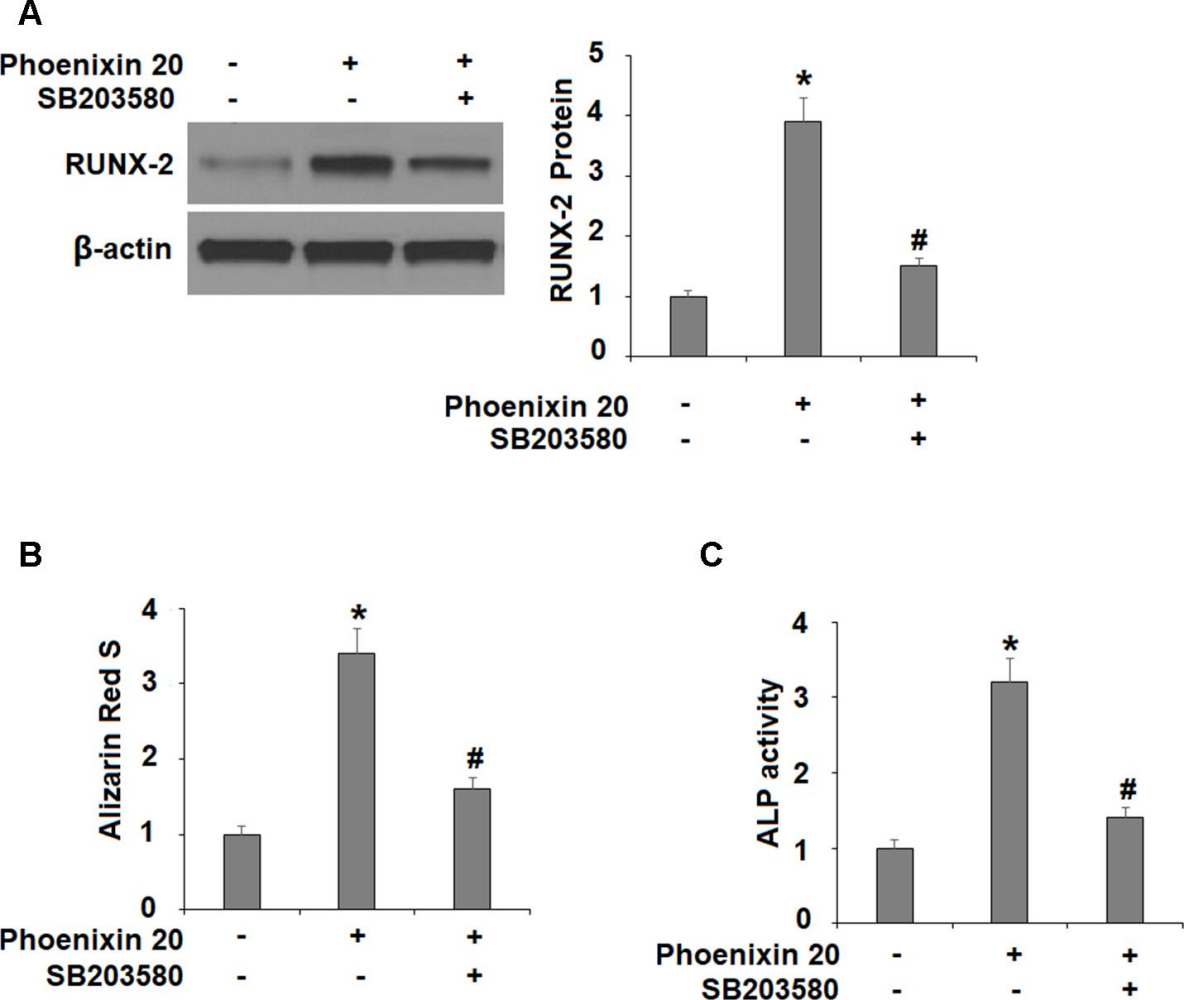
**The effects of Phoenixin 20 in osteoblast differentiation is mediated by p38.** Cells were cultured in osteogenic medium containing phoenixin 20 (15 nM) with or without SB203580 (10μM) for 14 days. (**A**) Protein of RUNX-2; (**B**) Alizarin Red S staining; (**C**) ALP activity (*, #, P<0.01, n=4).

The p38 MAPK kinase is important for osteoblastic differentiation. Compared to non-treated cells, two hours in the osteogenic media with phoenixin 20 induced a significantly greater increase in the amount of p-p38 (Figure 5). To verify the involvement of p38 in phoenixin 20-mediated osteoblast differentiation, we added the p38-specific inhibitor SB203580 in our 14-day differentiation experiment. The experimental results show that as compared to normal media, the induction of RUNX-2 transcripts was reduced by about 50% when SB203580 (10 μM) [16] was added to the media supplemented with phoenixin 20 (Figure 6A). Alizarin Red S staining demonstrated that matrix mineralization from the cells in media with SB203580 was less than half of that observed in the normal media (Figure 6B). The cells treated with SB203580 had about 505% ALP activity (Figure 6C).

### Phoenixin 20-mediated osteoblastic differentiation is dependent on GPR173

**Figure 7:**
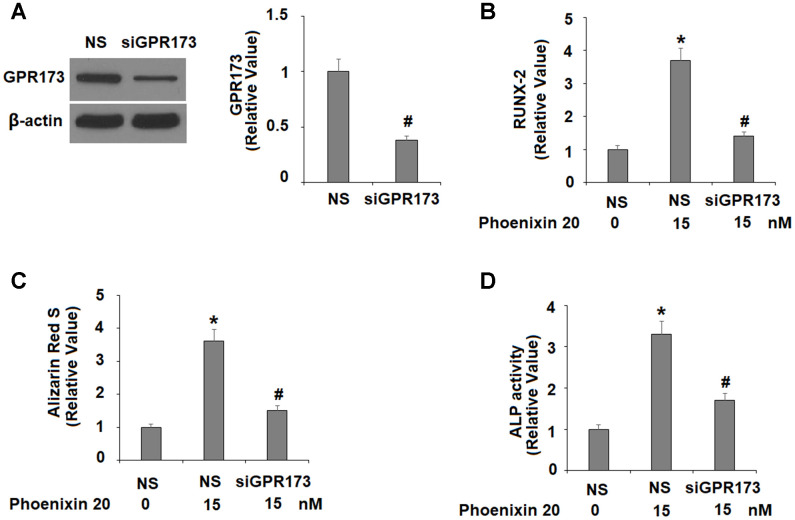
**The effects of phoenixin 20 in osteoblast differentiation are dependent on GPR173.** Cells were transfected with siGPR173 or non-specific siRNA; then cultured in osteogenic medium containing phoenixin 20 (15 nM) with for 14 days. (**A**) Successful knockdown of GPR173; (**B**) Levels of RUNX-2; (**C**) Alizarin Red S staining; (**D**) ALP activity (*, #, P<0.01, n=4-5).

Finally, we silenced GPR173 in MC3T3-E1 cells and evaluated the effects of its loss of function on phoenixin 20-mediated osteoblastic differentiation. Our western blot experiment confirmed that GPR173 was silenced by more than 60% after transfection with specific siRNA (Figure 7A). Compared to scramble cells, phoenixin 20-mediated osteoblastic differentiation was significantly weakened in GPR173-silent cells. RUNX-2 induction (Figure 7B), matrix mineralization (Figure 7C), and ALP activity (Figure 7D) were each reduced by about half in knockdown cells as compared to scramble cells.

## DISCUSSION

Osteoblasts are bone-building cells, and the fine regulation of osteogenic differentiation is critical to the process of bone formation, modeling, and remodeling. The understanding of signaling pathways involved in osteogenic differentiation may result in the discovery of potential targets of osteoporosis. The most common risk factors for osteoporosis include age, menopause-associated hormone changes in women, changes in physical activity, drugs, and other diseases [17, 18]. It is widely accepted that age-associated growth hormone, estrogens, and other hormones play a key role in the maintenance of bone homeostasis and the development of osteoporosis [19, 20]. Phoenixin represents a class of newly identified secreted peptides which influence neural and reproductive function in mammals [11, 12]. In 2013, a Stanford group successfully identified this peptide and dubbed it phoenixin [14]. Since then, collaborative efforts have led to the discovery of two major cleavage isoforms of phoenixin: a 20 amino acid peptide named phoenixin-20 and an N-terminally truncated form with 14 amino acid named phoenixin-14. The same group also reports that phoenixin 20 and phoenixin 14 have very similar biological actions [21]. However, both phoenixin 20 and phoenixin 14 were recently isolated from diverse tissues, including the brain, heart, lung, and stomach, suggesting that this class of peptides could have diverse functions [22].

In our effort to study the role of GPR173 in osteoblastic differentiation, we first noticed that mouse osteoblastic precursor MC3T3-E1 cells expressed abundant GPR173 transcripts and protein. This expression level is comparable to that in brain tissue, which is known to be high. Most intriguingly, our temporal assessment of GPR173 expression during osteoblast differentiation revealed that GPR173 could be induced in a time-dependent manner. These observations raised our interest to explore the possible role of this receptor in osteoblastic differentiation. Indeed, our study of GPR173 demonstrated that altering the expression of this receptor could influence multiple osteoblastic markers as well as their biological functions. We utilized the newly identified GPR173 agonist phoenixin 20 to raise the expression of GPR173 during osteoblastic differentiation. This peptide promoted the secretion of several osteoblastic signature genes, including ALP, Col-I, Osx, and OCN. Phoenixin 20 also promoted bone matrix formation as evidenced by increased matrix mineralization and ALP activity. These findings suggest that the activation of GPR173 promotes osteogenic differentiation. On the other hand, silencing of GPR173 largely reduces phoenixin 20-mediated osteoblastic differentiation. These findings suggest that phoenixin 20-mediated GPR173 signaling promotes osteogenic differentiation. Mechanistically, we show that the function of phoenixin 20 requires p38, as blockage of p38 by its inhibitor SB203580 attenuated much of its influence. Also, our knockdown experiment showed that the expression of GPR173 is required for the action of phoenixin 20, suggesting phoenixin 20 activity is strongly dependent on its receptor. Our finding is in agreement with a contemporary study showing that p38 mediates GPR173 signaling in immortalized neuron cells [23]. Therefore, the MAPK kinase pathway may serve as a common mediator of GPR173 signaling in different types of cells. p38 MAPK has been reported to play an essential role in different steps of osteoblast differentiation. In vivo experiments have shown that deficiency of p38 hampers osteoblast terminal differentiation and the appearance of osteocytes, which directly affects bone composition and maintenance [24]. As a matter of fact, p38 has been considered as a central hub for signaling convergence toward osteoblastogenesis due to its key action in bone development and homeostasis [25].

Metabolic control of bone remodeling is influenced by systematic hormonal regulation and local factors. The rate of remodeling is determined by loading and endocrine influences. Endocrine hormones, neural transmitters, and peptides produced by the CNS deliver signaling molecules to the environment surrounding bone cell surface receptors and thus influence bone remodeling [26]. Our study indicates that phoenixin 20, a newly recognized reproductive or neuronal hormone, could be another candidate for a neuronal regulator of bone metabolism.

So far, the signaling mediators of phoenixin 20 and its receptor GPR173 remain largely unknown, and our finding demonstrates that phoenixin 20-GPR173 signaling could play important roles in the regulation of bone remodeling. The main challenge in stem cell-based osteoporosis therapy is the direction of differentiation. How this ligand and receptor achieve regulatory function in bone metabolism and how they may interact with other signaling pathways to fine tune specific biological event would be of great interest for future studies, as they are potentially druggable molecular targets for treating various physiological diseases. Additionally, we only used an *in vitro* cell model in the current study. Future study using animal models will shed light on its potential therapeutic implications for osteoporosis.

## MATERIALS AND METHODS

### Cell culture

Mouse preosteoblast cell line MC3T3-E1 cells (Subclone 4) were from ATCC. Cells were maintained with DMEM (Gibco, USA) supplemented with 10% FBS (Gibco, USA), 10 mM HEPES (Sigma-Aldrich, USA), and 0.1% penicillin-streptomycin (Sigma Aldrich, USA) [27].

### Osteogenic differentiation

The differentiation of cultured MC3T3-E1 cells was induced by incubating osteogenic medium containing BMP-2, FBS (5%), β-glycerophosphate (3 mM), and ascorbic acid (50 μg/mL) for 3, 7, 14 days [28]. Cells maintained in normal growth media were used as the control.

### RT-PCR and real-time PCR

Cells were collected for gene expression profiling related to osteogenic differentiation. To perform qRT-PCR, RNA was isolated using Trizol (Invitrogen, USA) [29]. cDNA was produced from 2 μg RNA with reverse transcriptase as described by the M-MLV manual (New England Biolabs). For RT-PCR, GPR173 primers were used. The relative quantity of GPR173 was normalized to GAPDH. The expression of RUNX-2, ALP, Col-I, Osx, and OCN was detected using qPCR with SYBR Green Mix Kits (Applied Biosystems, USA). All results were quantitated using the 2^-ΔΔCt^ relative quantification method. The primer sequences were included in Table 1.

**Table 1:** Primer sequences.

	**Upstream Sequence (5’-3’)**	**Downstream Sequence (5’-3’)**
GAPDH	ACTGGCGTC TTCACCACCAT	AAG GCC ATG CCA GTG AGCTT
GPR173	CTTGACCTCCTCGGAAGACACTC	CGCCCACCACCTTGTAGCC
ALP	CTTGACCTCCTCGGAAGACACTC	CGCCCACCACCTTGTAGCC
RUNX-2	CACTGGCGGTGCAACAAGA	TTTCATAACAGCGGAGGCATTTC
OCN	GAGTCTGACAAAGCCTTCATGTCC	TGATAGCTCGTCACAAGCAGGGTTA
Osx	ATGGCGTCCTCTCTGCTTG	TGAAAGGTCAGCGTATGGCTT
Col-I	CCCAGAGTGGAACAGCGATT	ATGAGTTCTTCGCTGGGGTG

### Western blot analysis

The whole-cell lysates were made by lysing MC3T3-E1 cells with RIPA buffer. The samples were subjected to 10% SDS PAGE gel and transferred to a PVDF membrane [30]. The membranes were blocked and probed with primary antibodies and a secondary antibody. The following antibodies were used in this study: GPR173 (Cat#PA5-102093, Invitrogen, 1:1000), RUNX2 (Cat# ab76956, Abcam, 1: 2000), β-actin (Cat#4967, Cell Signaling Technology, 1:10000), p38 MAPK (Cat #9212, Cell Signaling Technology, 1:2000), Phospho-p38 MAPK (Thr180/Tyr182) (Cat #9211, Cell signaling Technology, 1:1000), goat anti-mouse IgG-HRP (Cat#sc-2005, Santa Cruz Biotechnology, 1:5000), mouse anti-rabbit IgG-HRP (Cat# sc-2357, Santa Cruz Biotechnology, 1:5000). After washing 3 times, the membrane was visualized to determine the activation response using HRP substrates (Thermo Fisher Scientific, USA). The membrane was exposed to X-ray film in a cassette (Kodak, USA).

### GPR173 knockdown

The siRNA target sequence was designed against mouse GPR173. For the knockdown experiment, 50% confluent MC3T3-E1 cells were transfected using Scrambled siRNA and designed GPR173-specific siRNAs using Lipofectamine RNAiMAX reagent (Invitrogen, USA). The efficiency of knockdown of GPR173 was confirmed 2 days after siRNA transfection.

### Alkaline phosphatase (ALP) activity

After reaching full confluence, the cells were equilibrated with ALPL buffer and incubated with 0.2 mg/mL nitro blue tetrazolium and 5-bromo-4-chloro-3-indolyl-phosphate (Sigma-Aldrich, USA). Cells were stained with ALPL buffer at room temperature for 2 hours. The fluorescent signal was measured using a microplate reader (405 nm).

### Alizarin Red S staining

Matrix mineralization was assessed through staining with Alizarin Red S. After stimulation, the cells were washed, followed by fixation with ethanol (70%) for 45 minutes. Cells were then stained with the dye Alizarin Red S. Fluorescence signals were visualized using a fluorescence microscope.

### Statistical analysis

Experiments were repeated for 3 times. Results are presented as means ± Standard Deviation (S.D.). The multiple group differences were assessed by analysis of variance (ANOVA) using the SPSS software (version 22.0), followed by the Bonferroni’s post-hoc test. A p-value < 0.05 was considered to be statistically significant.

## References

[1] Miller PD. Management of severe osteoporosis. Expert Opin Pharmacother. 2016; 17:473–88. 10.1517/14656566.2016.1124856 26605922

[2] Brown C, McKee C, Bakshi S, Walker K, Hakman E, Halassy S, Svinarich D, Dodds R, Govind CK, Chaudhry GR. Mesenchymal stem cells: cell therapy and regeneration potential. J Tissue Eng Regen Med. 2019; 13:1738–55. 10.1002/term.2914 31216380

[3] Du M, Pan W, Duan X, Yang P, Ge S. Lower dosage of aspirin promotes cell growth and osteogenic differentiation in murine bone marrow stromal cells. J Dent Sci. 2016; 11:315–22. 10.1016/j.jds.2016.03.009 30894990 PMC6395233

[4] Ducy P, Schinke T, Karsenty G. The osteoblast: a sophisticated fibroblast under central surveillance. Science. 2000; 289:1501–04. 10.1126/science.289.5484.1501 10968779

[5] Bustos F, Sepúlveda H, Prieto CP, Carrasco M, Díaz L, Palma J, Lattus J, Montecino M, Palma V. Runt-related transcription factor 2 induction during differentiation of wharton’s jelly mesenchymal stem cells to osteoblasts is regulated by jumonji AT-rich interactive domain 1B histone demethylase. Stem Cells. 2017; 35:2430–41. 10.1002/stem.2704 28895234

[6] Li Y, Kim JH, Choi EH, Han I. Promotion of osteogenic differentiation by non-thermal biocompatible plasma treated chitosan scaffold. Sci Rep. 2019; 9:3712. 10.1038/s41598-019-40371-6 30842578 PMC6403376

[7] Chen G, Deng C, Li YP. TGF-β and BMP signaling in osteoblast differentiation and bone formation. Int J Biol Sci. 2012; 8:272–88. 10.7150/ijbs.2929 22298955 PMC3269610

[8] Golub EE, Boesze-Battaglia K. The role of alkaline phosphatase in mineralization. Curr Opin Orthop. 2007; 18:444–48. 10.1097/BCO.0b013e3282630851

[9] Valenti MT, Dalle Carbonare L, Mottes M. Osteogenic differentiation in healthy and pathological conditions. Int J Mol Sci. 2016; 18:41. 10.3390/ijms18010041 28035992 PMC5297676

[10] Matsumoto M, Beltaifa S, Weickert CS, Herman MM, Hyde TM, Saunders RC, Lipska BK, Weinberger DR, Kleinman JE. A conserved mRNA expression profile of SREB2 (GPR85) in adult human, monkey, and rat forebrain. Brain Res Mol Brain Res. 2005; 138:58–69. 10.1016/j.molbrainres.2005.04.002 15893849

[11] Mcilwraith EK, Belsham DD. Phoenixin: uncovering its receptor, signaling and functions. Acta Pharmacol Sin. 2018; 39:774–78. 10.1038/aps.2018.13 29671415 PMC5943909

[12] Schalla MA, Stengel A. The role of phoenixin in behavior and food intake. Peptides. 2019; 114:38–43. 10.1016/j.peptides.2019.04.002 30953667

[13] Schalla MA, Stengel A. Phoenixin-a pleiotropic gut-brain peptide. Int J Mol Sci. 2018; 19:1726. 10.3390/ijms19061726 29891773 PMC6032287

[14] Yosten GL, Lyu RM, Hsueh AJ, Avsian-Kretchmer O, Chang JK, Tullock CW, Dun SL, Dun N, Samson WK. A novel reproductive peptide, phoenixin. J Neuroendocrinol. 2013; 25:206–15. 10.1111/j.1365-2826.2012.02381.x 22963497 PMC3556183

[15] Czekanska EM, Stoddart MJ, Richards RG, Hayes JS. In search of an osteoblast cell model for in vitro research. Eur Cell Mater. 2012; 24:1–17. 10.22203/ecm.v024a01 22777949

[16] Han X, Chen H, Zhou J, Steed H, Postovit LM, Fu Y. Pharmacological inhibition of p38 MAPK by SB203580 increases resistance to carboplatin in A2780cp cells and promotes growth in primary ovarian cancer cells. Int J Mol Sci. 2018; 19:2184. 10.3390/ijms19082184 30049957 PMC6121386

[17] Liu J, Curtis EM, Cooper C, Harvey NC. State of the art in osteoporosis risk assessment and treatment. J Endocrinol Invest. 2019; 42:1149–64. 10.1007/s40618-019-01041-6 30980341 PMC6751157

[18] Compston JE, McClung MR, Leslie WD. Osteoporosis. Lancet. 2019; 393:364–76. 10.1016/S0140-6736(18)32112-3 30696576

[19] Manolagas SC, O’Brien CA, Almeida M. The role of estrogen and androgen receptors in bone health and disease. Nat Rev Endocrinol. 2013; 9:699–712. 10.1038/nrendo.2013.179 24042328 PMC3971652

[20] Locatelli V, Bianchi VE. Effect of GH/IGF-1 on bone metabolism and osteoporsosis. Int J Endocrinol. 2014; 2014:235060. 10.1155/2014/235060 25147565 PMC4132406

[21] Stein LM, Haddock CJ, Samson WK, Kolar GR, Yosten GL. The phoenixins: from discovery of the hormone to identification of the receptor and potential physiologic actions. Peptides. 2018; 106:45–48. 10.1016/j.peptides.2018.06.005 29933026 PMC6092957

[22] Lyu RM, Cowan A, Zhang Y, Chen YH, Dun SL, Chang JK, Dun NJ, Luo JJ. Phoenixin: a novel brain-gut-skin peptide with multiple bioactivity. Acta Pharmacol Sin. 2018; 39:770–73. 10.1038/aps.2017.195 29542680 PMC5943905

[23] McIlwraith EK, Loganathan N, Belsham DD. Regulation of Gpr173 expression, a putative phoenixin receptor, by saturated fatty acid palmitate and endocrine-disrupting chemical bisphenol a through a p38-mediated mechanism in immortalized hypothalamic neurons. Mol Cell Endocrinol. 2019; 485:54–60. 10.1016/j.mce.2019.01.026 30716364

[24] Suzuki A, Guicheux J, Palmer G, Miura Y, Oiso Y, Bonjour JP, Caverzasio J. Evidence for a role of p38 MAP kinase in expression of alkaline phosphatase during osteoblastic cell differentiation. Bone. 2002; 30:91–98. 10.1016/s8756-3282(01)00660-3 11792570

[25] Rodríguez-Carballo E, Gámez B, Ventura F. P38 MAPK signaling in osteoblast differentiation. Front Cell Dev Biol. 2016; 4:40. 10.3389/fcell.2016.00040 27200351 PMC4858538

[26] Jones KB, Mollano AV, Morcuende JA, Cooper RR, Saltzman CL. Bone and brain: a review of neural, hormonal, and musculoskeletal connections. Iowa Orthop J. 2004; 24:123–32. 15296219 PMC1888423

[27] Wang C, Sun H, Zhong Y. Notoginsenoside R1 promotes MC3T3-E1 differentiation by up-regulating miR-23a via MAPK and JAK1/STAT3 pathways. Artif Cells Nanomed Biotechnol. 2019; 47:603–09. 10.1080/21691401.2019.1573189 30831034

[28] Xu X, Qiu S, Zhang Y, Yin J, Min S. PELA microspheres with encapsulated arginine-chitosan/pBMP-2 nanoparticles induce pBMP-2 controlled-release, transfected osteoblastic progenitor cells, and promoted osteogenic differentiation. Artif Cells Nanomed Biotechnol. 2017; 45:330–339. 10.3109/21691401.2016.1153480 26961803

[29] Zhu GZ, Zhang M, Kou CZ, Ni YH, Ji CB, Cao XG, Guo XR. Effects of Lyrm1 knockdown on mitochondrial function in 3 T3-L1 murine adipocytes. J Bioenerg Biomembr. 2012; 44:225–32. 10.1007/s10863-012-9404-9 22249831

[30] Zhang P, Ma J, Gao J, Liu F, Sun X, Fang F, Zhao S, Liu H. Downregulation of monocarboxylate transporter 1 inhibits the invasion and migration through suppression of the PI3K/Akt signaling pathway in human nasopharyngeal carcinoma cells. J Bioenerg Biomembr. 2018; 50:271–81. 10.1007/s10863-018-9763-y 29882205

